# Progressive Metabolic Dysregulation from Prediabetes to Diabetes Revealed by Untargeted Metabolomics

**DOI:** 10.3390/ijms27156721

**Published:** 2026-07-27

**Authors:** Maxat Toishimanov, Ivan Voitsekhovskiy, Aksholpan Shokan, Bakyt Kanapiyanov, Nurgul Myrzabayeva, Alma Nurtazina

**Affiliations:** 1 Kazakhstan-Japan Innovative Center, Kazakh National Agrarian Research University, Almaty 050010, Kazakhstan; 2 Faculty of Biology and Biotechnology, Al-Farabi Kazakh National University, Almaty 050038, Kazakhstan; 3 Department of Epidemiology and Biostatistics, Semey Medical University, Semey 071400, Kazakhstan; alma.nurtazina@smu.edu.kz; 4 Institute of Genetic and Physiology, Almaty 050040, Kazakhstan; 5 Department of Propaedeutics of Internal Diseases, Semey Medical University, Semey 071400, Kazakhstan; kebakyt@gmail.com

**Keywords:** untargeted metabolomics, prediabetes, diabetes, metabolic dysregulation, multivariate analysis

## Abstract

Type 2 diabetes (T2D) and prediabetes represent a progressive glycemic continuum associated with multi-pathway metabolic deterioration that often precedes clinical diagnosis. Early identification of molecular alterations underlying this transition is critical for prevention strategies. Untargeted gas chromatography–mass spectrometry (GC–MS) metabolomics combined with multivariate statistical analysis (PCA) was applied to serum samples from 188 participants stratified into Control (*n* = 48), Prediabetes (*n* = 113), and Diabetes (*n* = 27) groups according to ADA/WHO diagnostic criteria. Progressive metabolic alterations were observed across the glycemic continuum. Several amino acid-, lipid-, and organic acid-related features showed nominal differences between the study groups. However, none of the detected metabolomic features remained statistically significant after false discovery rate (FDR) correction, indicating that these findings should be considered exploratory. Diabetes was associated with widespread downregulation of amino acid-related features, long-chain and complex lipid species, and small organic acids relative to both control and prediabetes groups. PCA (PC1 = 35.9%) showed progressive metabolic stratification primarily driven by hyperglycemia, dyslipidemia, and blood pressure elevation. These exploratory findings suggest that the transition from prediabetes to diabetes may be accompanied by alterations in amino acid, lipid, and energy metabolism. The identified metabolomic features represent candidate metabolites that require validation in larger independent cohorts using targeted metabolomics.

## 1. Introduction

Type 2 diabetes (T2D) has emerged as one of the defining public health challenges of the twenty-first century. According to the 10th edition of the IDF Diabetes Atlas, 537 million adults were living with diabetes in 2021, with projections reaching 783 million by 2045 [1]. The recently released 11th edition updates the 2024 global estimate to 589 million (11.1%) and forecasts 853 million cases by 2050—a 44.8% increase within a single generation [2]. Parallel to overt diabetes, hundreds of millions of individuals live with impaired fasting glucose and impaired glucose tolerance, a silent prediabetic reservoir from which clinical T2D continuously emerges [1].

Before meeting diagnostic thresholds for T2D, most future patients traverse a period of metabolic syndrome (MetS)—a cluster of central obesity, atherogenic dyslipidemia, hypertension, and insulin resistance unified by chronic low-grade inflammation [3]. MetS is not simply a risk marker but a mechanistic precursor: insulin resistance, adipose-tissue dysfunction, and disturbed adipokine and cytokine signaling progressively exhaust pancreatic β-cell compensation and drive hyperglycemia [3]. The high prevalence of MetS in the prediabetes and diabetes groups of the present cohort (60% and 89%, respectively) underscores the clinical relevance of this overlap.

Despite the scale of the prediabetic population, routine diagnosis still relies on fasting plasma glucose (FPG) and glycated hemoglobin (HbA1c). These indices reflect sustained hyperglycemia rather than the early metabolic derangements that precede it and substantially misclassify individuals in the pre-disease window, missing early β-cell dysfunction and subtle glucose dysregulation [4]. There is a pressing need for molecular readouts capable of detecting dysmetabolism before overt glycemic failure and guiding earlier preventive intervention.

Untargeted metabolomics has emerged as a powerful discovery platform to address this diagnostic gap. Prospective meta-analyses across more than 70,000 participants confirm that circulating branched-chain and aromatic amino acids, acylcarnitines, diacylglycerols, and ceramides predict incident T2D, while glycine, glutamine, betaine, and lysophosphatidylcholines are inversely associated with risk [5]. Contemporary reviews describe how NMR- and MS-based profiling integrates with classical risk scores to enable precision prevention [6]. Among MS platforms, gas chromatography–mass spectrometry (GC–MS) is uniquely suited for polar primary metabolites—including amino acids, organic acids, simple sugars, and fatty acids—owing to robust electron-ionization spectral libraries, superior chromatographic resolution, and quantitative reproducibility [7,8]. Optimized GC–MS workflows have been successfully applied to characterize amino acid and microbiota-dependent metabolites in prediabetes and T2D sera [8].

An increasingly influential conceptual framework positions T2D and MetS as manifestations of accelerated biological aging [9,10]. The updated hallmarks of aging—mitochondrial dysfunction, cellular senescence, deregulated nutrient sensing, chronic inflammaging, epigenetic drift, and altered intercellular communication—map directly onto the pathophysiology of MetS and T2D [9,11]. Human-tissue studies demonstrate that senescent cells accumulate in adipose tissue, liver, and pancreatic β-cells in individuals with obesity and T2D independently of chronological age, sustained by hyperinsulinemia and reinforcing insulin resistance through the senescence-associated secretory phenotype (SASP) [10,11,12]. The insulin–IGF-1 signaling axis sits at the molecular interface between metabolism and longevity, with its dysregulation driving mTOR/FOXO imbalance that shortens health span and elevates T2D risk [13]. Cardiometabolic senescence burden further converges across atherosclerosis, diabetic cardiomyopathy, and heart failure, consolidating the cardiometabolic-aging continuum [14]. In this framework, prediabetes represents an intermediate stage of accelerated metabolic aging, and its circulating metabolome is expected to harbor early molecular echoes of aging biology.

Despite the expanding global literature, metabolomic data from Central Asian populations remain limited, despite the rapidly increasing prevalence of T2D across the region [15,16]. Furthermore, relatively few studies have examined metabolomic alterations across the full continuum from normoglycemia to prediabetes and established T2D within the same population. Therefore, the present study applied untargeted GC–MS serum metabolomics to 188 participants from Semey, Kazakhstan, stratified into Control (*n* = 48), Prediabetes (*n* = 113), and Diabetes (*n* = 27) groups, to explore metabolomic alterations across the glycemic continuum. We further explored whether the observed metabolomic alterations shared similarities with metabolic patterns previously associated with biological aging. Rather than identifying validated biomarkers or establishing shared molecular mechanisms, the present study was designed to identify exploratory candidate metabolomic features that may inform future targeted validation and longitudinal investigations.

## 2. Results

### 2.1. Baseline Characteristics of the Study Population

Baseline characteristics of the study population are presented in Table 1. A total of 188 participants were stratified into the Control (*n* = 48), Prediabetes (*n* = 113), and Diabetes (*n* = 27) groups based on ADA/WHO glycemic criteria.

Baseline clinical and biochemical characteristics of the study population are summarized in Table 1. Overall, participants with diabetes exhibited a progressively less favorable cardiometabolic profile than those with prediabetes and controls. Age, body weight, BMI, waist circumference, systolic and diastolic blood pressure, fasting glucose, HbA1c, total cholesterol, LDL cholesterol, and triglyceride concentrations increased significantly across the glycemic continuum (all *p* < 0.05), whereas HDL cholesterol decreased progressively. No significant differences were observed in height (*p* = 0.12) or smoking status (*p* = 0.21). The prevalence of hypertension and metabolic syndrome also increased markedly from the control group to prediabetes and diabetes (both *p* < 0.001). Detailed values for all clinical and biochemical parameters are presented in Table 1.

**Figure 1:**
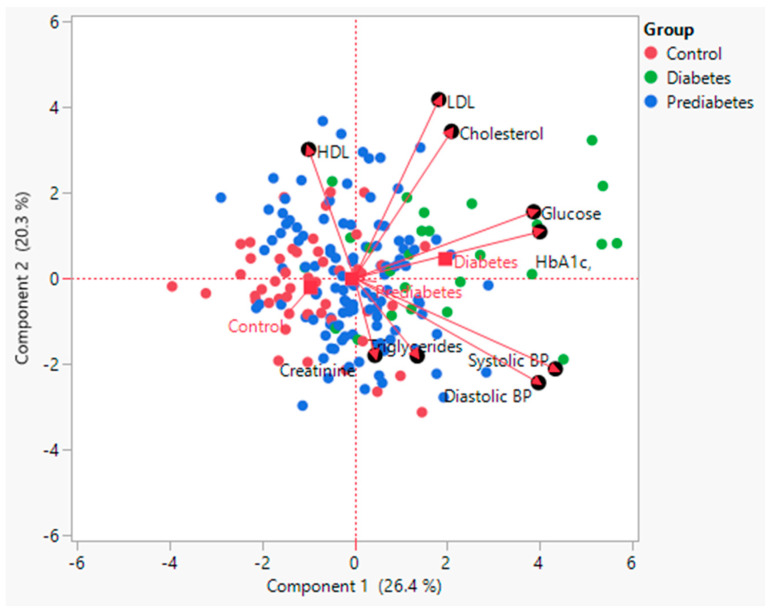
PCA score plot showing biochemical profile alterations among control, prediabetes, and diabetes groups.

The PCA biplot revealed trends in the association between clinical variables and the distribution of samples across the three study groups (Figure 1). The first principal component (PC1, 26.4%) primarily separated the diabetes group from the control group, while the second component (PC2, 20.3%) contributed to additional variation within and between groups.

The diabetes group tended to shift toward the positive side of PC1 and was strongly associated with elevated levels of glucose and HbA1c, as indicated by the direction and magnitude of the loading vectors. These variables showed the strongest contribution to group separation, highlighting hyperglycemia as the dominant metabolic feature of diabetes.

In addition, the diabetes cluster was aligned with increased low-density lipoprotein (LDL), total cholesterol, and triglycerides, reflecting significant lipid metabolism dysregulation and an atherogenic profile. The vectors for systolic and diastolic blood pressure were also oriented in the same direction, indicating a contribution of cardiovascular risk factors to the metabolic differentiation of the diabetes group.

In contrast, the control group was generally located in the negative region of PC1 and showed closer association with high-density lipoprotein (HDL), which was oriented in the opposite direction to LDL and cholesterol. This pattern reflects a protective lipid profile and metabolic homeostasis in healthy individuals.

The prediabetes group occupied an intermediate position, with partial overlap between control and diabetes clusters. This group showed weaker associations with glucose and HbA1c compared to diabetes but demonstrated emerging alignment with lipid-related variables, suggesting early-stage metabolic imbalance involving both glycemic and lipid pathways.

Creatinine was oriented toward the lower region of the plot, indicating its contribution to variability along PC2 and suggesting a secondary role related to renal function changes, rather than primary group discrimination.

Overall, the PCA biplot demonstrates that group separation is driven by a combination of hyperglycemia (glucose, HbA1c), dyslipidemia (LDL, cholesterol, triglycerides), blood pressure, and renal function markers, confirming that the progression from control to prediabetes and diabetes is associated with multifactorial metabolic and clinical disturbances.

### 2.2. MS-DIAL Feature Distribution

**Figure 2:**
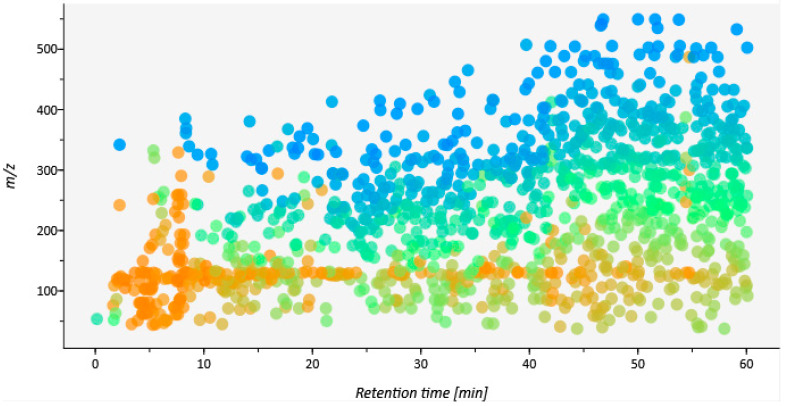
Distribution of detected features in MS-DIAL analysis plotted as mass-to-charge ratio (*m*/*z*) versus retention time. Colors represent feature density and retention time-dependent clustering patterns. Orange points correspond predominantly to early-eluting low-*m*/*z* compounds. Green and blue points represent progressively higher-*m*/*z* features detected at intermediate and late retention times.

The MS-DIAL feature distribution plot illustrates the global landscape of detected metabolites across the chromatographic run, showing the relationship between mass-to-charge ratio (*m*/*z*) and retention time (RT) (Figure 2). A wide range of features was detected, spanning RT values from approximately 0 to 60 min and *m*/*z* values from ~50 to over 500, indicating broad coverage of chemically diverse compounds.

Distinct clustering patterns were observed along the RT axis, reflecting differences in compound polarity and chemical class. Early eluting features (RT < 10 min) were predominantly located in the lower *m*/*z* range and likely correspond to highly polar metabolites, including small organic acids, amino acids, and other low-molecular-weight compounds. In contrast, features detected at intermediate retention times (10–40 min) showed a broader *m*/*z* distribution, suggesting the presence of semi-polar metabolites, such as intermediate metabolic products and small lipid-related molecules.

At longer retention times (RT > 40 min), features were more widely distributed toward higher *m*/*z* values, indicating the detection of less polar and higher-molecular-weight compounds, including lipid-related metabolites and complex organic molecules. This pattern reflects the chromatographic separation of compounds based on hydrophobicity, with more hydrophobic metabolites eluting later in the run.

The overall distribution demonstrates a high density of detected features in the mid-to-late retention time regions, suggesting that a substantial proportion of the metabolome is composed of moderately to weakly polar compounds. Additionally, the broad spread of *m*/*z* values across all retention times indicates the presence of structurally diverse metabolites within the dataset.

Untargeted GC–MS analysis revealed a number of significantly altered metabolic features between the studied groups. A total of differential metabolites were detected based on the criteria of VIP > 1.0, *p* < 0.05, and fold change (log2FC) values. Since no validation with authentic standards was performed, the detected compounds were reported as *m*/*z*–retention time features with putative chemical classification using MS-DIAL [17,18].

Compared with the control group, diabetes was characterized by widespread downregulation of amino acid-related compounds, organic acids, and lipid-related metabolites. Among the most affected features were amino acid-related metabolites (Features 370 and 291), lipid-related metabolites (Features 953 and 678), and several low-molecular-weight organic acids (Features 639 and 208), all showing significant decreases (Table 2).

Prediabetes exhibited a similar but less pronounced metabolic profile, with significant reductions primarily affecting amino acid-related metabolites and selected organic acids (Features 549, 245, and 589). Direct comparison between diabetes and prediabetes further demonstrated significant decreases in several long-chain and complex lipid-related features (Features 1221, 846, and 1263), indicating progressive metabolic alterations with disease severity.

The metabolomic profiling revealed coordinated reductions in amino acid-, organic acid-, and lipid-related metabolites in diabetes, whereas similar but less extensive changes were already detectable in prediabetes. Detailed information on all differential features is provided in Table 2.

### 2.3. Multivariate Analysis

PCA was performed as an unsupervised exploratory approach to visualize the overall structure of the metabolomic dataset. Although partial clustering of the three study groups was observed, considerable overlap remained, indicating that the global metabolic profiles were only moderately different. The first two principal components explained a substantial proportion of the total variance, with PC1 accounting for 35.9% and PC2 for 8.99%.

**Figure 3:**
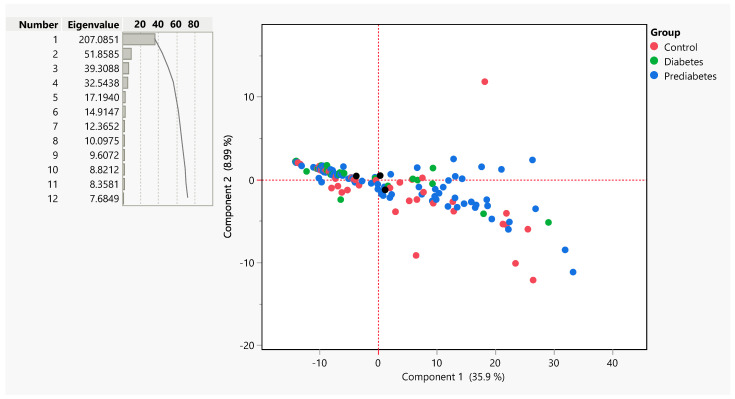
PCA score plot showing metabolic variation among control, prediabetes, and diabetes groups. Colored points represent individual samples from the three study groups (Control, Prediabetes, and Diabetes), whereas black points represent metabolite variables (loadings) contributing to the principal components.

As shown in Figure 3, a trend of separation was observed primarily along PC1, where samples from the diabetes group tended to shift toward higher positive values compared to the control group. In contrast, samples from the prediabetes group were distributed between control and diabetes clusters, indicating an intermediate metabolic phenotype. Although partial overlap among groups was present, the overall distribution pattern suggests gradual metabolic alterations associated with disease progression rather than distinct clustering.

The PCA loading plot indicated that a large number of variables contributed to the variance explained by PC1, suggesting that the observed separation is driven by coordinated changes across multiple metabolic features rather than a limited number of dominant metabolites. This observation is consistent with the results of univariate statistical analysis, which revealed multiple metabolites with moderate but significant differences between groups.

PCA results demonstrate that diabetes is associated with a global metabolic shift characterized by subtle but systematic alterations across multiple biochemical pathways, reflecting the complex and multifactorial nature of the disease.

### 2.4. Multiple-Testing Correction and Age-Adjusted Analysis

To improve the robustness of the statistical analysis and account for multiple comparisons, Benjamini–Hochberg false discovery rate (FDR) correction was applied to all quality-filtered metabolomic features. Among the 128 features included in the primary analysis, 21 demonstrated nominal statistical significance in the one-way ANOVA (*p* < 0.05). However, after correction for multiple testing, none remained statistically significant at the predefined threshold (q < 0.05). The complete feature-level statistical results, including both nominal *p*-values and FDR-adjusted q-values, are provided in Supplementary Table S1, whereas the metabolite features discussed in the manuscript are summarized in Supplementary Table S2.

Because the study groups differed significantly in age, an additional ANCOVA was performed using glycemic status as the fixed factor and age as a continuous covariate. Following adjustment, 20 metabolomic features remained nominally significant (ANCOVA, *p* < 0.05), whereas none remained significant after Benjamini–Hochberg correction (q < 0.05). Adjustment for age resulted in only minor changes in the ranking of the most discriminative metabolomic features, indicating that the observed metabolic variation was influenced by both glycemic status and age-related metabolic changes.

Accordingly, the biological interpretation presented in the following sections is based primarily on the age-adjusted analysis and the overall metabolomic patterns observed in the multivariate analyses rather than on nominal statistical significance of individual metabolites alone. The identified metabolites should therefore be considered exploratory candidate metabolites, requiring independent validation in larger cohorts and targeted metabolomic studies before their potential biological or clinical relevance can be confirmed.

## 3. Discussion

The present study applied untargeted GC–MS-based metabolomics combined with multivariate statistical approaches to characterize the metabolic landscape along a well-defined glycemic continuum in a Kazakh ethnic clinical cohort. Our findings suggest coordinated alterations in amino acid-, lipid-, and organic acid-related metabolomic features across the glycemic continuum. These results are consistent with and extend the current global metabolomics literature on T2D pathogenesis [5,19].

The downregulation of features tentatively classified as amino acid-related compounds—including features at *m*/*z* 130.0439, 122.1145, and 148.1869—is consistent with established alterations in amino acid metabolism in T2D. Depletion of glycine- and serine-like metabolites aligns with growing evidence linking hypoglycinemia to insulin resistance and incident T2D [20]. Glycine plays multiple metabolic roles: as an insulin secretagogue through pancreatic glycine receptors, as a precursor for glutathione synthesis, and as a buffer against oxidative stress; its depletion in the diabetic state reflects both increased utilization for antioxidant defense and diversion toward gluconeogenesis under conditions of impaired insulin signaling [20,21]. The differential metabolomic analysis suggests that amino acid-related metabolism may be altered during the progression from normoglycemia to diabetes. However, because the present study was based on untargeted metabolomics, the underlying molecular signaling pathways cannot be directly inferred from these data. Further targeted metabolomic and functional studies are required to elucidate the biological mechanisms responsible for these metabolic alterations [5,19,22].

One of the most striking findings was the pronounced downregulation of long-chain and complex lipid features in diabetes compared with both controls and prediabetes. Features tentatively annotated as long-chain lipids (e.g., *m*/*z* 301.2737, 438.1762, 485.6608) showed the largest fold-changes in the Diabetes versus Control and Diabetes versus Prediabetes comparisons. Although superficially counterintuitive given the hypertriglyceridemia documented in our clinical data, this pattern is consistent with dysregulated lipid processing and lipotoxicity mechanisms. On its face, this is counterintuitive, because the same participants displayed a progressively atherogenic clinical lipid profile, with total cholesterol (4.9 to 5.8 mmol/L), LDL cholesterol (2.7 to 3.6 mmol/L), and triglycerides (1.1 to 2.3 mmol/L) all rising across the glycemic continuum while HDL cholesterol declined (Table 1). Rather than resolve this apparent contradiction by appeal to mechanism, we note that the two observations are not directly comparable, because they derive from different measurements of different analytes. Clinical triglycerides were quantified by a standard enzymatic assay of total serum triglyceride content, whereas the GC–MS features represent derivatized, low-molecular-weight and free lipid-derived species; this platform does not detect or quantify intact triacylglycerols or phospholipids. A reduction in specific free or derivatizable lipid features can therefore coexist with elevated total circulating triglycerides—for example, if lipid is preferentially esterified into triacylglycerol, packaged into VLDL and other lipoprotein particles, or taken up by tissue rather than remaining as free species accessible to this method. We therefore regard this analytical and pre-analytical explanation as the most parsimonious account of the discrepancy, and we caution against over-interpreting the direction of individual lipid features obtained from an untargeted, semi-quantitative platform. The lipotoxicity mechanisms commonly invoked to explain insulin resistance in T2D—accumulation of diacylglycerols (DAGs) and ceramides, activation of novel PKC isoforms, and inhibition of insulin-receptor and Akt/PKB signaling [23,24]—were not measured in the present study and cannot be inferred from these data. Our untargeted design neither resolved these bioactive lipid species nor assessed their downstream signaling, so they are cited here only as biological context and as hypotheses to be tested, not as explanations of the feature-level changes we observed. Likewise, although prospective lipidomic and sphingolipid-profiling studies have described shifts in long-chain lipid and sphingolipid species across the dysglycemic continuum [25,26], those studies used targeted, quantitative platforms and are not directly equivalent to the putatively annotated features detected here. The GC–MS platform used here enables broad metabolite coverage but does not resolve intact phospholipid and sphingolipid species at the structural level; future integration of LC–MS/MS-based targeted lipidomics would enable precise quantification of these bioactive species.

The significant reduction in organic acid-related features in the diabetes group is consistent with impaired mitochondrial oxidative metabolism, particularly disruption of tricarboxylic acid (TCA) cycle flux. TCA cycle intermediates including citrate, succinate, and α-ketoglutarate are key nodes connecting fuel oxidation to cellular energy and biosynthetic demands; their perturbation reflects mitochondrial dysfunction increasingly recognized as a primary driver—rather than merely a consequence—of insulin resistance and T2D [27]. Succinate in particular has been proposed to play a pathogenic role through succinate receptor (SUCNR1)-mediated inflammatory macrophage recruitment and HIF-1α stabilization, contributing to metabolic inflammation in T2D [10]. The reduction in small organic acid features observed in our study (e.g., *m*/*z* 204.5174, RT 2.78 min) may additionally reflect depletion of α-hydroxybutyrate (α-HB), an early-stage biomarker of insulin resistance arising from elevated oxidative stress and altered NADH/NAD+ ratios under conditions of mitochondrial lipid overload [28]. These patterns collectively suggest metabolic inflexibility—the progressive inability to efficiently switch between fuel substrates—as a central feature of the glycemic progression continuum [29].

The PCA results confirmed progressive metabolic stratification across the three groups. PC1 (35.9% variance) primarily separated diabetes from controls, while the prediabetes group occupied an intermediate position with partial overlap—consistent with its biological status as a transitional metabolic state. This hierarchical separation in supervised multivariate analysis is consistent with findings from other population-based metabolomics studies characterizing the T2D continuum [5,19,30]. This clustering of metabolic syndrome components further supports the interpretation that the observed metabolomic alterations reflect systemic rather than pathway-specific metabolic disturbances.

The present study has several strengths, including a well-characterized clinical cohort with comprehensive biochemical profiling, systematic GC–MS data processing using MS-DIAL 4 [17], and application of complementary multivariate statistical approaches including PCA. Limitations include the cross-sectional design, the relatively modest size of the diabetes group (*n* = 27), and the absence of structural confirmation of metabolite identities using authentic chemical standards, which restricts annotation to putative levels (MSI levels 2–3) [31]. The GC–MS platform is optimized for volatile, semi-volatile, and derivatizable metabolites but provides limited coverage of intact polar lipid species, which are best quantified by LC–MS/MS-based lipidomics [32]. The study population reflects a Central Asian cohort from Semey, Kazakhstan; generalizability to other ethnic populations may be limited and warrants investigation. Future work should include targeted validation of key metabolites with authentic standards, expanded longitudinal cohorts to assess progression trajectories, and integration of multi-platform metabolomics to achieve broader metabolome coverage. Applying machine-learning-based classification approaches to the identified metabolite panels may further enhance predictive power for T2D risk stratification in clinical practice.

The exploratory metabolomic alterations observed in the present study may be consistent with metabolic patterns previously reported in metabolomic aging studies, including changes in amino acid-, organic acid-, and lipid-related metabolites. However, because the present study was cross-sectional and no metabolomic features remained statistically significant after FDR correction, these observations should be regarded as hypothesis-generating rather than evidence of shared biological mechanisms [33,34,35,36,37,38]. Because the present design is cross-sectional and untargeted, we interpret this overlap as a hypothesis-generating association rather than evidence of a shared mechanism: the metabolites that mark accelerated metabolic aging also change with glycemic progression, but we did not measure aging phenotypes, senescence markers, or their signaling.

Although the present findings do not support the identification of validated biomarkers because none of the individual metabolomic features remained statistically significant after FDR correction, the observed metabolomic alterations may still have potential clinical relevance. Rather than serving as standalone diagnostic biomarkers, combinations of candidate metabolomic features integrated with established clinical risk factors, such as age, BMI, fasting glucose, and HbA1c, may improve future risk stratification of individuals with prediabetes. Such multimarker approaches could help identify individuals who are more likely to progress to type 2 diabetes or, conversely, maintain stable glycemic control or revert to normoglycemia. However, this hypothesis requires confirmation in prospective longitudinal cohorts using targeted metabolomics and external validation before any clinical application can be considered.

This study has several limitations that should be considered when interpreting the findings. First, although several metabolomic features showed nominal statistical significance, none remained significant after FDR correction. Therefore, the reported metabolomic features should be considered exploratory candidate metabolites rather than validated biomarkers and require confirmation in larger independent cohorts using targeted metabolomics. Second, the relatively small and unbalanced diabetes group may have reduced the statistical power to detect significant metabolomic differences after correction for multiple testing and may limit the generalizability of the findings. Third, because this was a cross-sectional study, causal relationships between metabolomic alterations and progression from prediabetes to type 2 diabetes cannot be established, and longitudinal studies are needed to determine whether these metabolomic patterns predict disease progression. Finally, detailed information regarding hypoglycemic therapy was not available for all participants. Consequently, the potential influence of antidiabetic medications on circulating metabolite profiles could not be evaluated and may have contributed to the observed metabolic variation. Future studies incorporating larger cohorts, longitudinal follow-up, comprehensive medication data, and targeted metabolomic validation will be important to confirm the biological and clinical relevance of the identified candidate metabolites.

## 4. Materials and Methods

### 4.1. Study Design and Population

A cross-sectional study design was employed using a previously established cohort of participants from Semey City, Kazakhstan. The cohort was originally recruited using a two-stage random sampling approach. In the first stage, five out of 40 general practices (GPs) were randomly selected. In the second stage, eligible participants were randomly chosen from each GP unit using computer-generated random numbers, resulting in approximately equal representation across sites. This was a cross-sectional observational study. The sample size was determined by the availability of eligible participants meeting the predefined inclusion and exclusion criteria during the study period, and no a priori sample size calculation was performed.

Participants were stratified into three groups (Control, Prediabetes, and Diabetes) based on established clinical and biochemical criteria, including fasting glucose and HbA1c levels.

### 4.2. Data Collection and Clinical Measurements

Demographic and clinical data were collected using standardized questionnaires, including information on smoking status, family history, and hereditary predisposition to cardiovascular diseases (CVD) and hypertension (HT). Anthropometric measurements were obtained following WHO, ESC, and ESH guidelines.

Height was measured using a standardized stadiometer (Seca GmbH & Co. KG, Hamburg, Germany), and body weight was recorded using a calibrated scale (Tanita Corporation, Tokyo, Japan). Body mass index (BMI) was calculated as weight (kg) divided by height squared (m^2^). Waist circumference was measured using a non-elastic measuring tape (Seca GmbH & Co. KG, Hamburg, Germany).

Blood pressure (BP) was assessed using the Korotkov method in accordance with ESC/ESH recommendations. Measurements were taken with participants in a seated resting position, and the average of two consecutive readings was recorded.

Information regarding comorbidities and medication use was obtained from medical records and participant interviews. All participant data were anonymized and stored using coded identifiers to ensure confidentiality.

### 4.3. Participants and Group Classification

A total of 188 participants were included in the study. Based on clinical and biochemical criteria, participants were stratified into three groups: Control (*n* = 48), Prediabetes (*n* = 113), and Diabetes (*n* = 27). Fasting plasma glucose (FPG) and glycated hemoglobin (HbA1c) levels were used for classification according to international diagnostic guidelines (e.g., ADA/WHO criteria).

The Control group included individuals with normal glucose metabolism (FPG < 5.6 mmol/L and HbA1c < 5.7%). Prediabetes was defined as impaired glucose regulation, characterized by FPG between 5.6 and 6.9 mmol/L and/or HbA1c between 5.7% and 6.4%. Diabetes was defined as FPG ≥ 7.0 mmol/L and/or HbA1c ≥ 6.5%, or a previously established clinical diagnosis of diabetes.

Participants receiving glucose-lowering therapy or with a documented history of diabetes were also classified into the Diabetes group regardless of current biochemical values.

### 4.4. Biochemical Analysis

Fasting venous blood samples were collected from all participants after an overnight fast (8–12 h). Blood samples were processed immediately by centrifugation at 3000 rpm for 10 min to obtain serum, which was subsequently aliquoted and stored at −80 °C until analysis.

All blood samples were collected in the morning after an overnight fasting period of at least 12 h by venous puncture. Following collection, samples were processed under standardized laboratory conditions.

Serum levels of total cholesterol (TC), low-density lipoprotein cholesterol (LDL-C), high-density lipoprotein cholesterol (HDL-C), triglycerides (TG), fasting glucose, glycated hemoglobin (HbA1c), and creatinine were quantified using validated clinical laboratory methods in accordance with the manufacturer’s instructions.

Blood pressure measurements were performed according to established clinical guidelines, and the diagnosis of hypertension was defined based on systolic blood pressure ≥ 140 mmHg and/or diastolic blood pressure ≥ 90 mmHg, or the use of antihypertensive medication.

All analyses were conducted under standardized laboratory conditions with appropriate internal quality control procedures. Calibration was performed using certified reference materials, and analytical performance was monitored using control samples to ensure accuracy and precision of measurements.

### 4.5. GC–MS Analysis and Data Processing

For GC–MS analysis, serum extracts were derivatized prior to injection to improve volatility and chromatographic stability. Briefly, dried extracts were treated with methoxyamine hydrochloride in pyridine, followed by silylation with MSTFA containing 1% TMCS. An internal standard was added to each sample before extraction to monitor analytical reproducibility and injection stability. Quality control samples were prepared by pooling equal aliquots from all study samples and were injected periodically throughout the analytical sequence to assess instrument stability and signal reproducibility. Metabolite annotation was performed using MS-DIAL based on retention time, mass spectral similarity, and comparison with reference spectral libraries. Only features with acceptable peak shape, reproducible detection in QC samples, and reliable library matching were retained for downstream analysis. Because authentic reference standards were not used for confirmation, the reported metabolites should be considered putatively annotated features. No internal standard was used in the present untargeted GC–MS workflow. Instead, analytical reproducibility was monitored using pooled quality control (QC) samples analyzed periodically throughout the analytical sequence, and all samples were processed under identical extraction, derivatization, and instrumental conditions.

Plasma profiling was performed using a Trace GC 1310 gas chromatograph coupled with a TSQ 8000 triple quadrupole mass spectrometer (Thermo Scientific, Austin, TX, USA), equipped with an AS 1310 autosampler. Chromatographic separation was achieved on a TG-5SilMS capillary column (30 m × 0.25 mm i.d., 0.25 μm film thickness; Thermo Scientific, Austin, TX, USA).

The oven temperature program was as follows: initial temperature of 50 °C held for 5 min, followed by a linear increase to 250 °C at a rate of 5 °C/min, and a final hold at 250 °C for 15 min. Helium (99% purity) was used as the carrier gas at a constant flow rate of 1.0 mL/min, with a triple helium gas purification system (Thermo Scientific, Singapore). The septum purge flow was maintained at 3 mL/min throughout the analysis.

The injector temperature was set at 280 °C, while the transfer line and ion source temperatures were maintained at 250 °C and 240 °C, respectively. The mass spectrometer operated under electron ionization (EI) at 70 eV in full scan mode over a mass range of *m*/*z* 30–550, with a scan time of 0.2 s. The detector voltage was set to 0.96 kV.

Instrument control, data acquisition, and processing were carried out using Xcalibur software (version 4.3; Thermo Scientific).

Metabolite annotation confidence was interpreted according to the recommendations of the Metabolomics Standards Initiative (MSI) [36]. Because metabolite identities were assigned based on spectral library matching without confirmation using authentic reference standards or MS/MS fragmentation, all reported annotations were considered putative identifications (MSI Level 3).

### 4.6. Statistical Analysis

Statistical analyses were performed using JMP Pro 17 (SAS Institute Inc., Cary, NC, USA). Continuous variables were expressed as mean ± standard deviation (SD), while categorical variables were presented as counts and percentages.

Differences between groups (Control, Prediabetes, and Diabetes) were evaluated using one-way analysis of variance (ANOVA) followed by Tukey’s post hoc test for multiple comparisons. Categorical variables were analyzed using the chi-square (χ^2^) test. A *p*-value < 0.05 was considered statistically significant.

Metabolomics data processing, including peak detection, deconvolution, and alignment, was conducted using MS-DIAL software [17,18]. The resulting feature matrix was further analyzed statistically using JMP Pro 17.

For metabolomics data, multivariate statistical analyses were conducted to explore differences in metabolic profiles. Principal component analysis (PCA) was applied as an unsupervised method to visualize clustering patterns and detect outliers. The importance of variables was assessed using variable importance in projection (VIP) scores.

Prior to statistical analysis, metabolomics data were log-transformed and Pareto-scaled to reduce heteroscedasticity and improve comparability. Features with excessive missing values or low signal intensity were excluded. Missing values were imputed using appropriate statistical methods.

Univariate analysis of metabolomic features was performed using Student’s *t*-test or ANOVA, depending on the comparison. Fold change (FC) values were calculated, and volcano plots were constructed based on log_2_ (FC) and −log_10_ (*p*-value) to identify significantly altered features. Multiple testing correction was performed using the false discovery rate (FDR) approach. Both nominal *p*-values and FDR-adjusted q-values are reported. Statistical significance after multiple-testing correction was defined as q < 0.05. Age-adjusted analyses were performed using analysis of covariance (ANCOVA), with glycemic status as the fixed factor and age as a continuous covariate.

## 5. Conclusions

This cross-sectional untargeted GC–MS metabolomics study identified exploratory metabolomic alterations associated with the transition from normoglycemia to prediabetes and type 2 diabetes. Differential changes were observed in amino acid-, lipid-, and organic acid-related metabolomic features, suggesting that metabolomic remodeling may begin during the early stages of glycemic progression. However, the observed patterns were heterogeneous rather than strictly progressive, and none of the individual metabolomic features remained statistically significant after Benjamini–Hochberg false discovery rate correction following age-adjusted analysis. Therefore, the identified metabolites should be regarded as exploratory candidate metabolites rather than validated biomarkers.

Although the present findings provide additional insight into metabolic alterations associated with prediabetes and type 2 diabetes, the cross-sectional study design, putative metabolite annotation, and limited sample size warrant cautious interpretation. The observed metabolomic changes may contribute to future studies aimed at identifying metabolite panels associated with glycemic progression, but their biological and clinical relevance requires confirmation in larger independent cohorts using targeted metabolomics and structural validation with authentic reference standards. Future longitudinal studies integrating metabolomics with clinical and biochemical variables will be essential to determine whether combinations of metabolite features can improve the prediction of progression from prediabetes to type 2 diabetes.

The exploratory metabolomic alterations observed in the present study, including changes in amino acid-, organic acid-, and lipid-related metabolomic features across the glycemic continuum, may be consistent with metabolic patterns previously reported in studies of biological aging, including mitochondrial dysfunction, cellular senescence, and impaired nutrient sensing [9,10,17]. However, because the present study was cross-sectional and none of the detected metabolomic features remained statistically significant after false discovery rate correction, these observations should be regarded as hypothesis-generating rather than evidence of shared biological mechanisms. If confirmed in larger longitudinal studies, metabolomics-based profiling may contribute to a better understanding of the relationship between glycemic progression and biological aging in vulnerable populations [16,33].

## Figures and Tables

**Table 1:** Baseline characteristics of the study population.

Parameter	Control	Prediabetes	Diabetes	*p*-Value
Age (years)	44.8 ± 7.9 ^c^	52.6 ± 6.8 ^b^	58.9 ± 6.5 ^a^	<0.001
Height (cm)	166.3 ± 8.1	165.8 ± 7.9	162.4 ± 6.8	0.12
Weight (kg)	70.2 ± 11.5 ^c^	79.4 ± 14.2 ^b^	92.8 ± 16.3 ^a^	<0.001
BMI (kg/m^2^)	25.4 ± 3.8 ^c^	29.1 ± 4.2 ^b^	33.6 ± 5.1 ^a^	<0.001
Waist circumference (cm)	88.7 ± 9.4 ^c^	99.6 ± 10.2 ^b^	112.4 ± 12.6 ^a^	<0.001
Systolic BP (mmHg)	112 ± 12 ^c^	128 ± 15 ^b^	146 ± 18 ^a^	<0.001
Diastolic BP (mmHg)	72 ± 8 ^c^	81 ± 10 ^b^	92 ± 12 ^a^	<0.001
Glucose (mmol/L)	4.7 ± 0.4 ^c^	5.5 ± 0.6 ^b^	9.8 ± 2.8 ^a^	<0.001
HbA1c (%)	5.1 ± 0.3 ^c^	6.0 ± 0.2 ^b^	8.6 ± 1.7 ^a^	<0.001
Total cholesterol, mmol/L	4.9 ± 0.8 ^c^	5.3 ± 0.9 ^b^	5.8 ± 1.1 ^a^	0.04
LDL, mmol/L	2.7 ± 0.6 ^c^	3.1 ± 0.7 ^b^	3.6 ± 0.9 ^a^	0.02
HDL, mmol/L	1.55 ± 0.32 ^a^	1.28 ± 0.28 ^b^	1.12 ± 0.25 ^c^	<0.01
Triglycerides, mmol/L	1.1 ± 0.4 ^c^	1.6 ± 0.8 ^b^	2.3 ± 1.2 ^a^	<0.01
Hypertension, *n* (%)	8 (18%)	56 (49%)	22 (82%)	<0.001
Smoking, *n* (%)	6 (14%)	22 (19%)	8 (28%)	0.21
Metabolic syndrome, *n* (%)	5 (11%)	68 (60%)	25 (89%)	<0.001

Values are presented as mean ± standard deviation (SD) for continuous variables and as *n* (%) for categorical variables. Continuous variables were compared using one-way analysis of variance (ANOVA) followed by Tukey’s post hoc multiple comparison test. Categorical variables were analyzed using the chi-square (χ^2^) test. Different superscript letters (a–c) within the same row indicate statistically significant differences among groups according to Tukey’s post hoc test (*p* < 0.05); values sharing the same superscript letter are not significantly different. BMI, body mass index; BP, blood pressure; HbA1c, glycated hemoglobin; LDL, low-density lipoprotein cholesterol; HDL, high-density lipoprotein cholesterol.

**Table 2:** Differential metabolites identified by GC–MS analysis with putative annotation.

Feature ID	RT (min)	*m*/*z*		Tentative Annotation	Regulation	*p*-Value	log2FC
370	10.8	130.0439	4-Aminobutyric acid	Amino acid-related compound	↓ Diabetes	<0.001	−2.68
678	10.55	217.8107	Palmitic acid fragment	Fatty acid derivative	↓ Diabetes	<0.001	−3.35
419	54.73	130.2301		Lipid fragment	↓ Diabetes	<0.01	−2.73
953	54.71	301.2737	Monounsaturated fatty acid derivative	Long-chain lipid	↓ Diabetes	<0.01	−2.72
639	2.78	204.5174	Lactic acid	Small organic acid	↓ Diabetes	<0.001	−3.44
291	3.35	122.1145	Alanine	Amino acid derivative	↓ Diabetes	<0.05	−1.48
208	4.38	101.7664	Glycolic acid	Small metabolite	↓ Diabetes	<0.05	−1.21
144	4.88	81.1811	Acetic acid derivative	Low-mass organic compound	↓ Diabetes	<0.05	−1.13
591	44.01	176.1621	Succinic acid	Organic acid/lipid fragment	↓ Diabetes	<0.05	−1.52
455	43.7	131.1557	Palmitic acid fragment	Fatty acid fragment	↓ Diabetes	<0.01	−1.86
549	8.19	148.1869	Valine	Amino acid	↓ Prediabetes	<0.01	−1.51
496	8.56	134.5953	Threonine	Amino acid derivative	↓ Prediabetes	<0.01	−1.77
166	8.46	85.9071	Pyruvic acid derivative	Small metabolite	↓ Prediabetes	<0.01	−1.67
526	9.01	140.0671	Glutamic acid derivative	Organic compound	↓ Prediabetes	<0.01	−1.69
589	7.65	174.1351	Fumaric acid	Organic acid	↓ Prediabetes	<0.05	−1.30
245	8.05	116.0372	Alanine	Amino acid	↓	<0.05	−1.48
218	7.88	107.0648	Glycine derivative	Amino acid fragment	↓ Prediabetes	<0.05	−1.42
1221	34.99	438.1762	Cholesterol-related lipid	Long-chain lipid	↓ Diabetes vs. PreD	<0.01	−1.58
846	34.96	277.3116	Linoleic acid	Lipid	↓ Diabetes vs. PreD	<0.05	−1.46
1238	48.88	457.7885	Sterol	Complex lipid	↓ Diabetes vs. PreD	<0.01	−1.95
1263	54.71	485.6608	Cholesteryl ester-related lipid	Long-chain lipid	↓ Diabetes vs. PreD	<0.01	−1.78

↓ indicates a decrease (downregulation) in the reported parameter.

## Data Availability

The raw data supporting the conclusions of this article are available in the Zenodo repository at https://doi.org/10.5281/zenodo.21393362.

## Supplementary Materials

The following supporting information can be downloaded at: https://www.mdpi.com/article/10.3390/ijms27156721/s1.
