# Supplementary material for: Progressive Metabolic Dysregulation from Prediabetes to Diabetes Revealed by Untargeted Metabolomics
Source: Int J Mol Sci. 2026 Jul 27;27(15):6721. doi: 10.3390/ijms27156721 (PMC13466226; doi:10.3390/ijms27156721)
Supplement: Supplementary file 1 [file ijms-27-06721-s001.zip › ijms-4399607-supplementary.pdf]

Supplementary Table S1. Complete feature-level statistical analysis of all quality-filtered metabolomic features obtained by untargeted GC–MS.

Feature ID	Tentative annotation	Ontology	RT (min)	m/z	Fill fraction	Average S/N	Mean log2 Control	Mean log2 Prediabetes	Mean log2 Diabetes	FC PD/C	FC D/C	FC D/PD	Raw p (ANOVA)	FDR q (ANOVA)	Age-adjusted ANCOVA p	Age-adjusted ANCOVA q	Pairwise p: PD/C	Pairwise p: D/C	Pairwise p: D/PD
20	Hexamethylene tetramine	1,3,5-triazinanes	22.813	36.067	0.8400	826.860	23.422	22.911	22.789	0.7016	0.6449	0.9193	0.5299	0.7559	0.5447	0.7688	0.2684	0.2938	0.8296
21	Cadaverine	Monoalkylamines	22.478	36.071	0.9600	259.690	22.444	21.883	21.422	0.6779	0.4924	0.7263	0.2911	0.5479	0.3324	0.6079	0.2175	0.1020	0.4266
22	DL-Coniine	Alkaloids and derivatives	12.499	36.076	0.9000	2078.700	23.629	23.098	22.943	0.6919	0.6214	0.8982	0.3911	0.6502	0.4155	0.6819	0.1664	0.2020	0.7653
44	Pyrazol	Pyrazoles	13.847	44.114	0.8500	279.930	22.393	21.985	21.537	0.7538	0.5526	0.7331	0.3807	0.6435	0.4327	0.7010	0.3250	0.1383	0.4081
76	N-Nitrosopiperidine (NPIP)	Nitrosopiperidines	28.927	57.193	0.8300	289.730	21.857	21.646	21.202	0.8639	0.6352	0.7352	0.6568	0.7931	0.5969	0.7889	0.6602	0.3242	0.4736
78	Cadaverine	Monoalkylamines	26.408	58.123	0.8100	303.240	21.698	21.511	21.327	0.8781	0.7732	0.8805	0.8740	0.9227	0.8437	0.8970	0.7092	0.5817	0.7668
79	5-Aminoimidazole-4-carboxamide	Aminoimidazoles	13.706	58.140	0.8800	285.560	22.439	21.870	21.484	0.6741	0.5159	0.7653	0.2610	0.5219	0.2809	0.5451	0.1615	0.0969	0.4774
92	N,N-Dimethylsulfamide	Sulfuric acid diamides	9.442	64.129	0.8500	12959.170	24.625	23.490	23.505	0.4552	0.4601	1.011	0.0650	0.3133	0.0621	0.3613	0.0152	0.1181	0.9814
93	Indole-3-carbinol	3-alkylindoles	5.077	64.332	0.8300	57683.270	30.992	30.027	29.958	0.5122	0.4882	0.9533	0.0235	0.2731	0.0273	0.3146	0.0123	0.0277	0.8597
96	Betaine aldehyde	Tetraalkylammonium salts	4.671	66.600	0.8000	20067.870	30.837	30.097	29.927	0.5988	0.5323	0.8891	0.0722	0.3133	0.0958	0.3834	0.0464	0.0416	0.6433
119	Gamma-Aminobutyric Acid_major	Gamma amino acids and derivatives	43.259	72.168	0.8200	198.000000	22.360	21.689	21.657	0.6278	0.6143	0.9786	0.3289	0.5767	0.2826	0.5451	0.1188	0.2663	0.9582
130	(1,1-Dimethylethyl)urea	Ureas	12.642	75.120	0.9200	1560.570	23.436	23.089	22.611	0.7859	0.5645	0.7183	0.3821	0.6435	0.3875	0.6614	0.3779	0.1670	0.3947

135	4-Piperidinecarboxamide	Piperidinecarboxamides	21.968	79.093	0.9400	1280.450	23.549	22.919	22.697	0.6463	0.5542	0.8574	0.3008	0.5580	0.2787	0.5451	0.1435	0.1459	0.6838
139	Indole-3-carbinol	3-alkylindoles	4.808	80.452	0.8000	16394.430	30.644	29.690	29.759	0.5160	0.5415	1.049	0.0425	0.2927	0.0873	0.3834	0.0145	0.0499	0.8574
142	Orcinol	Resorcinols	9.549	81.090	0.8200	1970.690	24.244	23.243	23.186	0.4998	0.4805	0.9613	0.0998	0.3753	0.0916	0.3834	0.0317	0.1092	0.9246
143	2-(Piperazin-1-yl)ethanamine	N-alkylpiperazines	11.562	81.158	0.8100	1141.260	23.772	22.846	22.318	0.5264	0.3650	0.6934	0.0551	0.3133	0.0773	0.3803	0.0486	0.0311	0.3764
144	Cadaverine	Monoalkylamines	4.883	81.181	0.8000	19734.770	31.004	30.062	29.870	0.5206	0.4558	0.8756	0.0179	0.2550	0.0197	0.2796	0.0129	0.0087	0.5810
148	2-(Piperazin-1-yl)ethanamine	N-alkylpiperazines	8.930	83.747	0.8300	67616.840	25.621	24.155	24.119	0.3620	0.3530	0.9752	0.0486	0.2959	0.0495	0.3165	0.0152	0.0908	0.9638
152	Pesticide1_Methamidophos_C2H8NO2PS_O,S-Dimethyl phosphoramidothioate	Phosphoramidothioic-acid-O,S-diesters	22.582	84.375	0.8000	853.280	22.941	22.748	22.982	0.8742	1.029	1.177	0.8876	0.9227	0.8870	0.9156	0.6824	0.9441	0.6630
157	2-(Piperazin-1-yl)ethanamine	N-alkylpiperazines	44.765	85.160	0.8800	354.920	23.526	22.907	22.623	0.6512	0.5348	0.8214	0.3172	0.5718	0.2791	0.5451	0.1595	0.1456	0.6281
159	3-Aminopropionitrile	Nitriles	25.932	85.164	0.8100	194.520	21.741	21.427	21.261	0.8041	0.7167	0.8913	0.7727	0.8676	0.7595	0.8454	0.5355	0.5008	0.8003
166	Styrene	Styrenes	8.461	85.907	0.8600	201526.400	29.758	28.081	27.890	0.3127	0.2739	0.8760	0.0069	0.2207	0.0145	0.2796	0.0018	0.0149	0.7826
168	Piperazine	Piperazines	12.790	87.091	0.8300	990.190	23.480	22.630	22.170	0.5546	0.4031	0.7269	0.0734	0.3133	0.0841	0.3834	0.0345	0.0379	0.4419
170	Octanal	Medium-chain aldehydes	12.110	88.181	0.8300	654.510	23.272	22.432	22.117	0.5584	0.4490	0.8041	0.1355	0.3753	0.1726	0.4433	0.0502	0.0542	0.5829
171	2-Methylpyrrolidine; CE0; RGHPCLZJAFCTIK-UHFFFAOYSA-N	Pyrrolidines	17.038	88.418	0.8500	458.560	22.974	22.389	22.328	0.6670	0.6395	0.9587	0.3101	0.5670	0.3478	0.6099	0.1175	0.1940	0.8959
201	2-(Piperazin-1-yl)ethanamine	N-alkylpiperazines	9.119	99.988	0.8100	6371.580	25.212	23.773	23.391	0.3688	0.2831	0.7674	0.0109	0.2207	0.0145	0.2796	0.0091	0.0196	0.5655

205	N-Methyl-L-proline; CE0; CWLQUGTUXBXTLF-YFKPBYRVSA-N	Proline and derivatives	3.637	100.984	0.8600	926235.600	32.315	31.409	31.176	0.5338	0.4543	0.8510	0.1452	0.3753	0.1742	0.4433	0.0632	0.1158	0.7243
216	2-Amino-2-(hydroxymethyl)-1,3-propanediol	1,2-aminoalcohols	17.159	105.543	0.9000	409.000000	22.441	21.839	21.421	0.6587	0.4929	0.7483	0.2121	0.4682	0.2239	0.5078	0.1404	0.0843	0.4489
219	Isoamylamine	Monoalkylamines	7.877	107.065	0.8100	16384.970	30.904	29.473	29.863	0.3707	0.4860	1.311	0.0094	0.2207	0.0126	0.2796	0.0024	0.0731	0.4422
223	Melamine	1,3,5-triazines	2.092	108.867	0.9000	############	31.773	30.929	30.985	0.5570	0.5791	1.040	0.0457	0.2927	0.0467	0.3146	0.0258	0.0876	0.8794
227	2-Aminophenol; CE0; CDAWCLOXVUBKRW-UHFFFAOYSA-N	Aniline and substituted anilines	22.205	110.830	0.8900	733.220	23.207	22.678	22.593	0.6931	0.6532	0.9425	0.4905	0.7474	0.4908	0.7661	0.2295	0.2985	0.8775
230	N-ACETYLPUTRESCINE	Carboximidic acids	14.744	111.262	0.8600	634.000000	22.928	22.088	21.699	0.5588	0.4268	0.7638	0.0841	0.3362	0.1028	0.3987	0.0438	0.0364	0.4722
231	5-METHYLCYTOSINE HYDROCLORIDE	Hydroxypyrimidines	14.947	112.168	0.9600	2668.380	24.135	23.353	23.276	0.5815	0.5514	0.9482	0.1450	0.3753	0.1441	0.4356	0.0403	0.1035	0.8778
234	Isoamylamine	Monoalkylamines	46.132	113.185	0.8500	174.470	22.321	21.692	21.381	0.6467	0.5213	0.8061	0.2467	0.5169	0.2421	0.5252	0.1229	0.1139	0.5805
239	Glutamine (D)	Alpha amino acids	27.899	113.237	0.8400	1561.600	23.632	23.554	23.481	0.9476	0.9009	0.9507	0.9763	0.9803	0.9646	0.9722	0.8716	0.8126	0.9013
241	Pesticide1_Methamidophos_C2H8NO2PS_O,S-Dimethyl phosphoramidothioate	Phosphoramidothioic-acid-O,S-diesters	8.656	114.821	0.8500	55313.210	27.901	26.281	26.087	0.3254	0.2845	0.8743	0.0326	0.2811	0.0451	0.3146	0.0099	0.0565	0.8228
243	Isoguvacine	Hydropyridines	12.911	115.174	0.9100	658.540	23.257	22.746	22.458	0.7014	0.5747	0.8194	0.3241	0.5762	0.3478	0.6099	0.2069	0.1426	0.5595
248	4-(Dimethylamino)pyridine	Dialkylarylamines	8.246	116.552	0.9000	169004.500	30.591	29.358	29.965	0.4255	0.6478	1.522	0.0337	0.2811	0.0414	0.3146	0.0109	0.2567	0.2074
257	Orcinol	Resorcinols	6.960	117.839	0.8300	537110.300	29.078	28.469	28.730	0.6559	0.7862	1.199	0.7223	0.8329	0.7189	0.8294	0.3989	0.7223	0.7726
262	5-Flurocytosine	Halopyrimidines	5.856	118.153	0.8600	109483.600	28.297	27.937	28.145	0.7791	0.8996	1.155	0.8295	0.8922	0.8225	0.8847	0.5623	0.8590	0.7826
264	Pyrazinamide	Pyrazines	13.489	119.107	0.8800	5251.690	23.892	23.333	23.112	0.6787	0.5823	0.8579	0.2727	0.5289	0.3157	0.5857	0.1256	0.1117	0.6334
279	Hexamethylene tetramine	1,3,5-triazinanes	10.447	120.317	0.9800	395507.500	29.488	28.411	28.809	0.4741	0.6246	1.318	0.1643	0.3894	0.1776	0.4433	0.0517	0.3489	0.5450

281	2-(Piperazin-1-yl)ethanamine	N-alkylpiperazines	11.460	120.801	0.8100	4303.840	24.729	23.710	23.390	0.4937	0.3954	0.8009	0.0622	0.3133	0.0934	0.3834	0.0235	0.0604	0.6276
295	Maltol	Pyranones and derivatives	2.194	122.212	0.9800	############	31.130	30.321	30.427	0.5711	0.6144	1.076	0.0824	0.3362	0.0713	0.3801	0.0315	0.1491	0.8015
297	Piperazine	Piperazines	11.852	122.535	0.9000	1247.720	23.841	23.016	22.508	0.5645	0.3972	0.7036	0.1194	0.3753	0.1296	0.4251	0.0943	0.0380	0.3720
303	1-PHENYLETHANOL	Benzene and substituted derivatives	2.323	123.963	0.9000	138380.200	29.124	28.430	28.771	0.6184	0.7830	1.266	0.2230	0.4800	0.2261	0.5078	0.0809	0.4375	0.4019
305	Creatinine	Alpha amino acids and derivatives	9.312	124.097	0.9600	4881.850	24.663	23.557	23.356	0.4648	0.4042	0.8694	0.0599	0.3133	0.0750	0.3803	0.0245	0.0591	0.7445
307	5,6-Dimethylbenzimidazole	Benzimidazoles	16.409	124.222	0.8000	856.610	21.543	21.052	20.351	0.7115	0.4378	0.6153	0.1540	0.3753	0.2317	0.5113	0.2607	0.0431	0.1808
309	2-(Piperazin-1-yl)ethanamine	N-alkylpiperazines	28.402	125.249	0.9100	593.440	22.883	22.598	22.432	0.8206	0.7314	0.8913	0.7867	0.8681	0.7939	0.8685	0.5712	0.5051	0.7842
313	ACETYLCHOLINE	Acyl cholines	14.814	126.237	0.9400	2456.840	23.499	23.001	23.122	0.7080	0.7697	1.087	0.5199	0.7559	0.5125	0.7677	0.2208	0.4964	0.8169
316	Octylamine	Monoalkylamines	10.928	126.280	0.9500	10943.690	24.307	23.378	23.008	0.5253	0.4063	0.7735	0.1188	0.3753	0.1605	0.4422	0.0705	0.0748	0.5685
318	1,3-Phenylenediamine	Aniline and substituted anilines	7.970	127.117	0.8600	17211.780	30.912	29.616	29.932	0.4071	0.5068	1.245	0.0166	0.2550	0.0194	0.2796	0.0068	0.0590	0.4508
319	Agmatine	Guanidines	22.317	127.136	0.8800	1931.750	23.537	23.257	23.481	0.8233	0.9620	1.169	0.8129	0.8818	0.8041	0.8723	0.5333	0.9160	0.6443
330	5,6-Dihydro-5-methyluracil	Hydropyrimidines	14.206	127.277	0.8500	############	29.155	28.028	28.171	0.4579	0.5055	1.104	0.1107	0.3753	0.1246	0.4251	0.0248	0.1846	0.8374
334	Fumaric acid	Dicarboxylic acids and derivatives	18.254	128.140	0.9400	365.310	23.402	22.610	22.414	0.5777	0.5043	0.8730	0.1487	0.3753	0.1992	0.4723	0.0509	0.0883	0.7196
337	Goitrin	Oxazolidines	10.277	128.209	0.8300	############	31.357	29.810	29.763	0.3422	0.3314	0.9684	0.0302	0.2811	0.0456	0.3146	0.0089	0.0495	0.9492

342	MEVALOLACTONE	Delta valerolactones	14.470	129.052	0.9500	2970.020	23.624	23.066	22.672	0.6794	0.5171	0.7611	0.2696	0.5289	0.2747	0.5451	0.1848	0.1066	0.4702
343	N-ACETYLPUTRESCINE	Carboximidic acids	20.809	129.060	0.9700	6702.060	25.465	25.065	24.784	0.7581	0.6237	0.8228	0.4575	0.7203	0.5120	0.7677	0.2833	0.2270	0.6015
344	5-Flurocytosine	Halopyrimidines	5.468	129.074	0.8200	152848.100	30.940	30.032	30.052	0.5328	0.5403	1.014	0.0351	0.2811	0.0394	0.3146	0.0296	0.0866	0.9594
345	5,6-Dihydro-5-methyluracil	Hydropyrimidines	20.917	129.086	0.9200	3020.360	25.285	24.948	24.678	0.7917	0.6566	0.8293	0.5795	0.7808	0.5022	0.7677	0.3918	0.2700	0.6082
346	Octylamine	Monoalkylamines	53.468	129.118	0.8300	218.910	22.050	21.272	20.594	0.5833	0.3646	0.6250	0.1319	0.3753	0.1094	0.4001	0.1282	0.0522	0.3227
348	5,6-Dihydro-5-methyluracil	Hydropyrimidines	1.726	129.142	0.9600	7955.970	24.460	23.562	23.310	0.5364	0.4505	0.8399	0.0678	0.3133	0.0596	0.3613	0.0343	0.0594	0.6431
350	Agmatine	Guanidines	44.617	129.144	0.9200	721.510	23.483	23.012	22.786	0.7216	0.6172	0.8552	0.4493	0.7203	0.4530	0.7248	0.2287	0.2236	0.6835
352	HISTIDINOL	Aralkylamines	23.095	129.162	0.8500	387.980	22.923	22.420	22.352	0.7058	0.6731	0.9537	0.5492	0.7641	0.6091	0.7956	0.2585	0.3586	0.9081
354	5,6-Dimethylbenzimidazole	Benzimidazoles	33.545	129.167	0.8000	305.010	21.906	21.604	21.250	0.8113	0.6347	0.7823	0.6667	0.7975	0.5979	0.7889	0.5490	0.3440	0.5787
355	Glycine	Alpha amino acids	1.627	129.170	1.000000	16680.100	25.475	24.806	24.422	0.6290	0.4821	0.7665	0.1053	0.3753	0.1329	0.4251	0.0490	0.0391	0.4337
356	Octanol	Fatty alcohols	28.170	129.176	0.9100	823.020	23.287	22.790	22.754	0.7088	0.6913	0.9753	0.5616	0.7729	0.5664	0.7796	0.2697	0.3986	0.9513
358	2-(Piperazin-1-yl)ethanamine	N-alkylpiperazines	14.108	129.185	0.8600	5994.280	24.188	23.677	24.055	0.7014	0.9118	1.300	0.5315	0.7559	0.5466	0.7688	0.2407	0.8398	0.5505
361	2-(Piperazin-1-yl)ethanamine	N-alkylpiperazines	15.593	129.225	0.9500	15925.430	26.456	25.925	25.883	0.6921	0.6721	0.9710	0.4569	0.7203	0.5541	0.7710	0.1759	0.3036	0.9375
363	5-Flurocytosine	Halopyrimidines	5.376	129.673	0.9200	398477.700	30.935	30.204	30.381	0.6024	0.6813	1.131	0.1397	0.3753	0.1286	0.4251	0.0657	0.1998	0.6004
364	Benzamidine	Benzene and substituted derivatives	13.558	129.693	0.8800	1798.670	23.221	22.358	22.039	0.5499	0.4407	0.8013	0.1142	0.3753	0.1275	0.4251	0.0505	0.0694	0.5962
365	N-Methyl-L-proline; CE0; CWLQUGTUXBXTLF-YFKPBYRVSA-N	Proline and derivatives	10.686	129.737	0.9200	62265.470	25.899	24.867	25.197	0.4887	0.6144	1.257	0.2521	0.5169	0.2630	0.5451	0.1003	0.4018	0.6552

366	Isophorone	Cyclohexenones	35.929	129.738	0.9400	549976.300	30.282	29.774	29.757	0.7034	0.6949	0.9879	0.4870	0.7474	0.6233	0.8059	0.2081	0.3944	0.9760
367	Octylamine	Monoalkylamines	16.509	129.922	0.9900	2443.400	24.059	23.382	23.147	0.6254	0.5313	0.8496	0.1554	0.3753	0.1865	0.4505	0.0530	0.0704	0.6285
368	Naphthalene	Naphthalenes	14.299	129.937	0.8300	48722.290	24.706	24.161	24.350	0.6854	0.7814	1.140	0.7109	0.8272	0.6659	0.8294	0.4135	0.6872	0.8081
370	Creatinine	Alpha amino acids and derivatives	16.359	129.968	0.9900	1582.550	23.533	22.718	22.830	0.5685	0.6143	1.081	0.1012	0.3753	0.1094	0.4001	0.0264	0.1795	0.8164
371	Isoguvacine	Hydropyridines	1.985	129.988	0.9700	############	30.857	29.839	29.989	0.4939	0.5480	1.110	0.0453	0.2927	0.0457	0.3146	0.0249	0.1347	0.7512
372	O-Acetylserine	L-alpha-amino acids	10.797	130.044	0.8700	21535.360	25.884	23.957	23.203	0.2631	0.1560	0.5929	0.0013	0.1661	0.0018	0.2248	0.0029	0.0008	0.2420
374	Indole-3-carbinol	3-alkylindoles	20.039	130.069	0.8000	33676.340	27.933	27.424	27.172	0.7024	0.5898	0.8397	0.5029	0.7485	0.5406	0.7688	0.2645	0.2303	0.6841
378	Goitrin	Oxazolidines	21.724	130.118	0.9700	2155.590	24.162	23.749	23.531	0.7513	0.6459	0.8598	0.5385	0.7575	0.5780	0.7871	0.3260	0.2581	0.6749
379	2-(Piperazin-1-yl)ethanamine	N-alkylpiperazines	17.940	130.128	0.9800	2865.210	23.945	23.288	23.112	0.6342	0.5616	0.8854	0.2544	0.5169	0.2995	0.5637	0.0921	0.1424	0.7473
385	Octylamine	Monoalkylamines	14.012	130.148	0.9500	3588.260	24.585	23.732	23.403	0.5535	0.4407	0.7962	0.0705	0.3133	0.0834	0.3834	0.0311	0.0365	0.5289
386	2-(Piperazin-1-yl)ethanamine	N-alkylpiperazines	30.005	130.150	0.9000	1357.720	23.044	22.650	22.288	0.7610	0.5920	0.7779	0.5097	0.7499	0.4735	0.7482	0.3614	0.2164	0.5374
387	1-(4-methoxyphenyl)ethanone	Alkyl-phenylketones	13.911	130.153	0.8300	416.300	22.055	21.292	20.935	0.5895	0.4601	0.7806	0.1534	0.3753	0.1577	0.4422	0.0684	0.0980	0.5771
400	4-Hydroxyphenylethanol	1-hydroxy-2-unsubstituted benzenoids	32.454	130.183	0.8100	395.040	21.817	21.762	21.172	0.9628	0.6395	0.6642	0.6323	0.7931	0.5415	0.7688	0.9169	0.3692	0.3584
403	Octylamine	Monoalkylamines	41.947	130.188	0.9700	5480.660	25.486	25.027	25.060	0.7274	0.7445	1.023	0.6032	0.7931	0.6470	0.8258	0.2474	0.5199	0.9589
405	Octylamine	Monoalkylamines	19.124	130.190	0.9800	6441.530	25.815	25.525	25.369	0.8177	0.7340	0.8977	0.7576	0.8582	0.8550	0.8970	0.4855	0.4382	0.7823
407	N-Nitrosopiperazine (NPAZ)	Piperazines	15.771	130.199	0.9000	2544.430	23.921	23.409	23.346	0.7011	0.6712	0.9574	0.4138	0.6790	0.3967	0.6681	0.1788	0.2612	0.8955

413	Octylamine	Monoalkylamines	17.808	130.203	0.8900	1275.290	23.799	23.173	22.910	0.6478	0.5398	0.8333	0.2250	0.4800	0.2853	0.5451	0.0965	0.0937	0.6044
415	5,6-Dihydro-5-methyluracil	Hydropyrimidines	13.215	130.211	0.8900	4223.140	24.367	23.711	23.257	0.6348	0.4633	0.7299	0.1771	0.3981	0.2708	0.5451	0.1010	0.0649	0.4324
416	Octylamine	Monoalkylamines	41.154	130.213	0.9000	573.550	22.770	22.431	22.179	0.7906	0.6636	0.8395	0.6394	0.7931	0.5456	0.7688	0.4628	0.3100	0.6296
420	Agmatine	Guanidines	24.002	130.229	0.9500	1968.930	24.317	23.891	23.762	0.7441	0.6803	0.9143	0.6318	0.7931	0.7417	0.8454	0.3280	0.3630	0.8287
423	6-Mercaptopurine	Purinethiones	33.006	130.231	0.8200	446.030	22.597	22.300	22.014	0.8144	0.6680	0.8202	0.7063	0.8272	0.7192	0.8294	0.5487	0.3908	0.6451
430	Octylamine	Monoalkylamines	48.110	130.244	0.8500	503.110	22.824	22.406	21.507	0.7481	0.4013	0.5364	0.1707	0.3973	0.1746	0.4433	0.3640	0.0602	0.1723
432	Octanal	Medium-chain aldehydes	44.998	130.252	0.8200	476.770	22.521	22.098	22.050	0.7458	0.7214	0.9673	0.6495	0.7931	0.6795	0.8294	0.3530	0.4674	0.9364
438	Tropinone	Tropane alkaloids	16.794	130.302	0.8900	254020.300	28.715	27.651	27.618	0.4782	0.4674	0.9774	0.1183	0.3753	0.1624	0.4422	0.0219	0.1043	0.9599
440	Itaconic acid	Branched fatty acids	21.439	131.087	0.8400	3133.160	24.654	24.245	24.132	0.7530	0.6964	0.9248	0.6327	0.7931	0.7033	0.8294	0.3333	0.3326	0.8295
441	beta-N-Methylaminoalanine	Alpha amino acids	13.392	131.090	0.8500	842.430	22.929	22.160	21.748	0.5869	0.4410	0.7516	0.1274	0.3753	0.1476	0.4356	0.0925	0.0682	0.4735
449	Agmatine	Guanidines	26.163	131.133	0.8000	405.770	22.360	22.223	21.907	0.9094	0.7304	0.8032	0.8120	0.8818	0.8508	0.8970	0.7655	0.4879	0.6118
455	L-ORNITHINE	L-alpha-amino acids	38.577	131.151	0.9000	542.230	22.396	22.033	22.134	0.7779	0.8339	1.072	0.7006	0.8272	0.6962	0.8294	0.3591	0.6207	0.8418
457	GUANIDINOACETATE	Alpha amino acids and derivatives	43.902	131.154	0.8100	783.720	22.687	22.054	20.861	0.6450	0.2822	0.4375	0.0646	0.3133	0.0663	0.3690	0.2360	0.0172	0.0870
462	Agmatine	Guanidines	37.611	131.160	0.8400	1290.430	23.711	23.323	23.310	0.7645	0.7575	0.9908	0.6559	0.7931	0.6901	0.8294	0.3449	0.4857	0.9801
465	N-ACETYLPUTRESCINE	Carboximidic acids	34.996	131.163	0.9900	4516.750	25.061	25.064	24.013	1.002	0.4838	0.4829	0.1773	0.3981	0.2241	0.5078	0.9952	0.0820	0.0630
480	2-(Piperazin-1-yl)ethanamine	N-alkylpiperazines	37.152	131.182	0.9600	1172.190	24.028	23.752	23.990	0.8261	0.9746	1.180	0.7827	0.8681	0.7829	0.8639	0.4855	0.9456	0.6475
483	BENZALDEHYDE	Benzoyl derivatives	27.184	131.198	0.8400	319.180	21.756	21.489	21.224	0.8312	0.6913	0.8318	0.7371	0.8424	0.7186	0.8294	0.5774	0.4221	0.6628

486	Octylamine	Monoalkylamines	23.318	131.200	0.9300	2978.110	24.549	23.900	24.027	0.6377	0.6966	1.092	0.3581	0.6194	0.3789	0.6554	0.1160	0.3406	0.8075
490	L-Threonine; CE0; AYFVYJQAPQTCCC-GBXIJSLDSA-N	L-alpha-amino acids	42.532	131.219	0.9800	24917.260	27.330	26.883	26.610	0.7337	0.6072	0.8276	0.4614	0.7203	0.5158	0.7677	0.2618	0.2351	0.6403
494	2,3-Dihydroxypyridine; CE0; GGOZGYRTNQBSSA-UHFFFAOYSA-N	Pyridinones	28.647	133.089	0.8000	275.450	22.261	22.058	21.947	0.8686	0.8044	0.9262	0.9010	0.9227	0.8834	0.9156	0.6989	0.6449	0.8580
497	N-ACETYL-L-LEUCINE	Leucine and derivatives	32.804	133.151	0.9100	878.770	22.935	22.696	22.585	0.8474	0.7843	0.9256	0.8462	0.9026	0.7536	0.8454	0.6156	0.5890	0.8497
500	Pesticide1_Methamidophos_C2H8NO2PS_O,S-Dimethyl phosphoramidothioate	Phosphoramidothioic-acid-O,S-diesters	8.561	134.595	0.8800	136508.300	28.863	27.075	27.020	0.2896	0.2788	0.9628	0.0121	0.2207	0.0161	0.2796	0.0017	0.0287	0.9445
506	Octylamine	Monoalkylamines	17.450	136.072	0.9600	2853.250	23.831	23.069	23.086	0.5895	0.5966	1.012	0.1531	0.3753	0.1425	0.4356	0.0426	0.1775	0.9731
507	1-Acetylimidazole	Carbonylimidazoles	34.281	136.122	0.9500	1963.100	23.482	23.397	23.147	0.9431	0.7929	0.8408	0.8958	0.9227	0.9380	0.9572	0.8632	0.6119	0.6823
510	L-ORNITHINE	L-alpha-amino acids	27.447	136.186	0.9700	2244.300	24.295	24.154	23.650	0.9071	0.6396	0.7051	0.6308	0.7931	0.7151	0.8294	0.7478	0.2942	0.3984
518	PHENYLETHANOLAMINE	Aralkylamines	36.299	138.167	1.000000	111391.100	27.491	27.407	27.445	0.9432	0.9686	1.027	0.9803	0.9803	0.9831	0.9831	0.8258	0.9357	0.9443
521	Dipropenyl disulfide	Organic disulfides	8.364	138.326	0.9000	250623.500	30.296	28.808	29.257	0.3566	0.4868	1.365	0.0209	0.2679	0.0295	0.3146	0.0037	0.1126	0.4544
523	Benzamide	Benzamides	2.690	138.690	0.8500	############	31.380	30.861	30.856	0.6982	0.6956	0.9964	0.2789	0.5329	0.3428	0.6099	0.2052	0.3084	0.9890
530	BETAINE	Alpha amino acids	9.012	140.067	0.9100	12991.790	25.488	23.799	23.891	0.3103	0.3308	1.066	0.0032	0.2042	0.0041	0.2611	0.0021	0.0296	0.8802
532	L-ASPARAGINE	Asparagine and derivatives	35.410	140.131	0.9000	9680.820	22.463	22.321	21.749	0.9061	0.6099	0.6730	0.6321	0.7931	0.5864	0.7889	0.7915	0.2540	0.3055
537	2-(Piperazin-1-yl)ethanamine	N-alkylpiperazines	48.871	140.306	0.8100	28543.000000	25.365	24.355	23.026	0.4966	0.1976	0.3979	0.0406	0.2927	0.0295	0.3146	0.1119	0.0166	0.1345
542	N-Acetylalanine	N-acyl-alpha amino acids	8.795	141.996	0.9000	31390.220	26.549	24.931	24.692	0.3257	0.2760	0.8475	0.0325	0.2811	0.0386	0.3146	0.0151	0.0591	0.7830

555	((1S,9aR)-octahydro-1H-quinolizin-1-yl)methanamine	Quinolizines	8.190	148.187	0.8000	47207.650	30.303	28.788	29.468	0.3498	0.5608	1.603	0.0052	0.2207	0.0078	0.2796	0.0009	0.0742	0.1064
558	Cyprodinil-TP CGA 249287	Aminopyrimidines and derivatives	29.912	150.170	0.9600	6230.780	24.226	24.027	23.944	0.8715	0.8226	0.9439	0.9136	0.9281	0.9423	0.9572	0.6696	0.6894	0.9050
560	Agmatine	Guanidines	36.454	151.158	0.9500	2260.070	23.876	23.763	23.202	0.9246	0.6268	0.6779	0.5917	0.7889	0.6516	0.8258	0.7931	0.2547	0.3351
568	Benzotriazole	Benzotriazoles	16.240	158.164	0.9400	11278.590	24.867	24.073	23.661	0.5771	0.4335	0.7511	0.1455	0.3753	0.1801	0.4433	0.0656	0.0516	0.4874
572	7-Hydroxyoctanoic acid	Medium-chain hydroxy acids and derivatives	16.079	160.212	0.8800	147532.500	27.623	27.103	27.005	0.6974	0.6518	0.9346	0.5684	0.7740	0.6802	0.8294	0.2750	0.3606	0.8799
577	5-Flurocytosine	Halopyrimidines	5.741	162.228	0.9000	235760.800	29.244	28.659	28.462	0.6669	0.5818	0.8724	0.5006	0.7485	0.4094	0.6806	0.2733	0.3036	0.7786
578	Octylamine	Monoalkylamines	18.063	162.319	0.8800	27029.500	25.189	24.154	24.090	0.4880	0.4671	0.9572	0.1335	0.3753	0.1498	0.4356	0.0296	0.1352	0.9288
1000	CP_M308c	Phenylpropanes	16.748	308.226	0.8500	8239.100	19.006	18.867	19.098	0.9075	1.066	1.174	0.8885	0.9227	0.7550	0.8454	0.6865	0.8878	0.7229

Supplementary Table S2. Exploratory candidate features discussed in the manuscript with nominal and FDR-adjusted statistical results

Feature ID	MS-DIAL annotation	Ontology	RT (min)	m/z	Fill fraction	Average S/N	FC PD/C	FC D/C	FC D/PD	Raw p (ANOVA)	FDR q (ANOVA)	Age-adjusted ANCOVA p	Pairwise p PD/C	Pairwise p D/C	Pairwise p D/PD
370	Creatinine	Alpha amino acids and derivatives	16.359	129.968	0.9900	1582.550	0.5685	0.6143	1.081	0.1012	0.3753	0.1094	0.0264	0.1795	0.8164
678	Meprobamate	Carbamate esters	16.958	217.437	0.1200	405.980	0.6941	0.5505	0.7932	0.3089		0.2732	0.1797	0.1146	0.5193

419	4-Fluoraniline	Aniline and substituted anilines	41.319	130.222	0.7100	320.960	0.6862	0.6455	0.9408	0.5024		0.5221	0.2378	0.3388	0.8869
953	(2E,4E)-N-(2-methylpropyl)dodeca-2,4-dienamide	N-acyl amines	45.628	299.703	0.3100	190.850	0.8261	0.5721	0.6925	0.4058		0.3683	0.4821	0.1615	0.3306
639	NCGC00180087-02!5-(hydroxymethyl)-3-(1-hydroxy-4-methylhexyl)oxolan-2-one [IIN-based: Match]	Fatty alcohols	51.050	204.153	0.4000	161.890	0.9752	0.7464	0.7654	0.6834		0.6083	0.9175	0.3743	0.3906
291	N-Nitrosopiperazine (NPAZ)	Piperazines	6.518	122.107	0.7500	18036.110	0.4728	0.5336	1.129	0.1724		0.1341	0.0664	0.2349	0.7931
208	Octylamine	Monoalkylamines	59.056	101.638	0.2800	67.930	0.6919	0.4802	0.6941	0.1697		0.1922	0.1476	0.0443	0.2947
144	Cadaverine	Monoalkylamines	4.883	81.181	0.8000	19734.770	0.5206	0.4558	0.8756	0.0179	0.2550	0.0197	0.0129	0.0087	0.5810
591	Ropivacaine	Alpha amino acid amides	34.205	173.141	0.1900	10379.400	1.150	1.157	1.006	0.9436		0.8210	0.7422	0.7930	0.9900
455	L-ORNITHINE	L-alpha-amino acids	38.577	131.151	0.9000	542.230	0.7779	0.8339	1.072	0.7006	0.8272	0.6962	0.3591	0.6207	0.8418
549	MENTHOL(-)	Menthane monoterpenoids	20.497	145.677	0.3400	104.310	0.7409	0.5253	0.7090	0.2655		0.2764	0.2882	0.0881	0.3071
496	2-Methylindole	Indoles	52.151	133.125	0.4800	82.760	0.7295	0.4692	0.6432	0.1706		0.1749	0.2317	0.0392	0.2016
166	Styrene	Styrenes	8.461	85.907	0.8600	201526.400	0.3127	0.2739	0.8760	0.0069	0.2207	0.0145	0.0018	0.0149	0.7826
526	N-tert-Butylisopropylamine	Dialkylamines	30.924	138.909	0.4800	10985.950	0.9581	0.4829	0.5040	0.3600		0.3440	0.9069	0.1364	0.1451
589	4-amino-1-((2R,3R,4S,5R)-3,4-dihydroxy-5-(hydroxymethyl)tetrahydrofuran-2-yl)pyrimidin-2(1H)-one	Pyrimidine nucleosides	9.423	172.272	0.3700	2852.970	0.5659	0.6625	1.171	0.2353		0.2737	0.0752	0.4001	0.7278

245	Aspartic acid	Aspartic acid and derivatives	6.373	116.028	0.6500	180928.200	0.5858	0.4465	0.7622	0.3553		0.3338	0.2426	0.2059	0.6254
218	1-(4-methoxyphenyl)ethanone	Alkyl-phenylketones	18.909	106.773	0.7100	395.450	0.5765	0.5584	0.9686	0.2046		0.2356	0.0671	0.1770	0.9367
1221	8-(azepan-1-ium-1-ylmethyl)-3-(2,4-dimethoxyphenyl)-2-oxo-2H-chromen-7-olate	Hydroxyisoflavonoids	27.541	432.180	0.0300	1408.870	1.174	0.7488	0.6379	0.3242		0.3658	0.4950	0.3169	0.0930
846	Venlafaxine	Anisoles	23.308	276.482	0.4800	26953.070	0.7410	0.7690	1.038	0.7084		0.7823	0.3743	0.5764	0.9333
1238	NCGC00380875-01_C21H32O11_4-(4-Hydroxyphenyl)-2-butanyl 6-O-[(4xi)-alpha-L-threo-pentofuranosyl]-beta-D-glucopyranoside	Fatty acyl glycosides of mono- and disaccharides	52.088	440.874	0.0400	203.200	1.374	0.8546	0.6221	0.2634		0.2306	0.2587	0.6715	0.1453
1263	NCGC00384804-01_C20H30O11_2-(4-Methoxyphenyl)ethyl 6-O-alpha-L-arabinopyranosyl-beta-D-glucopyranoside	O-glycosyl compounds	33.811	465.360	0.0700	121.450	1.358	0.7555	0.5562	0.1103		0.1085	0.2488	0.4245	0.0489
